# Autophagy in Crohn’s Disease: Converging on Dysfunctional Innate Immunity

**DOI:** 10.3390/cells12131779

**Published:** 2023-07-04

**Authors:** Kibrom M. Alula, Arianne L. Theiss

**Affiliations:** Division of Gastroenterology & Hepatology, University of Colorado Anschutz Medical Campus, Aurora, CO 80045, USA

**Keywords:** Crohn’s disease, innate immunity, mitophagy, xenophagy, ER stress, mitochondria, ATG16L1, NOD2, IRGM, inflammatory bowel disease, genetic susceptibility

## Abstract

Crohn’s disease (CD) is a chronic inflammatory bowel disease marked by relapsing, transmural intestinal inflammation driven by innate and adaptive immune responses. Autophagy is a multi-step process that plays a critical role in maintaining cellular homeostasis by degrading intracellular components, such as damaged organelles and invading bacteria. Dysregulation of autophagy in CD is revealed by the identification of several susceptibility genes, including *ATG16L1*, *IRGM*, *NOD2*, *LRRK2*, *ULK1*, *ATG4*, and *TCF4*, that are involved in autophagy. In this review, the role of altered autophagy in the mucosal innate immune response in the context of CD is discussed, with a specific focus on dendritic cells, macrophages, Paneth cells, and goblet cells. Selective autophagy, such as xenophagy, ERphagy, and mitophagy, that play crucial roles in maintaining intestinal homeostasis in these innate immune cells, are discussed. As our understanding of autophagy in CD pathogenesis evolves, the development of autophagy-targeted therapeutics may benefit subsets of patients harboring impaired autophagy.

## 1. Introduction

Crohn’s disease (CD) is a chronic inflammatory bowel disease (IBD) affecting the gastrointestinal tract in which the immune system attacks healthy tissue, leading to transmural inflammation and damage. As of 2020, over 5 million people in North America and Europe, and millions more worldwide, are affected by IBD [1]. The symptoms of CD can vary widely from person to person, but may include abdominal pain, diarrhea, weight loss, fatigue, and fever. Some people may also experience extra-intestinal inflammation affecting the joints, eyes, or other locations, and/or complications, such as bowel obstruction, fistulas, or abscesses, attesting to the heterogenous nature of this disease. Inflammation in CD can occur in any part of the gastrointestinal tract from the mouth to the anus, but it most commonly affects the ileum and colon. CD inflammation is characterized by infiltration of immune cells, such as macrophages, neutrophils, and activated effector Th1 and Th17 cells coupled with decreased T regulatory (Treg) cell activity [2,3]. In addition, CD can present hallmark granulomas, which are clusters of white blood cells that form in response to chronic inflammation [4]. IBD is associated with intestinal microbiota dysbiosis, defined as a loss of homeostatic gut microorganism composition and non-beneficial interaction with host epithelial and immune cells [5].

The etiology of CD is not known, but it is thought to be a combination of dysregulated immune response, genetic, and environmental factors. There is currently no cure for CD, but treatment options, such as medication, surgery, and dietary changes, can help manage symptoms and improve quality of life for people with the disease [6]. Anti-inflammatory medications, immunosuppressants, and biologics are commonly used to reduce inflammation and prevent further damage to the intestinal tissue. Despite these treatment options, 80% of patients require surgery to remove damaged tissue or to treat complications, such as strictures or fistulas, over the course of their disease [7]. In addition, current treatment options suffer from the heterogenous nature of Crohn’s with a noted biologic ceiling of response across patients [8]. Genome-wide association studies (GWAS) have provided important insights into the genetic contribution to IBD susceptibility, with novel disease mechanisms revealed, including CD risk alleles converging on autophagy [9]. Although identification of susceptibility alleles provides an associative link for involvement in CD, later studies have demonstrated functional alterations of genetic polymorphisms and impact on the autophagy pathway and intestinal cell physiology. Other genes related to the susceptibility of CD include *IL23R* (immune response and inflammation) [10], *SLC39A8* (manganese homeostasis and intestinal barrier integrity) [11], *CARD9* (microbial sensing and handling) [12], *ECM1* and *HNF4A* (maintenance of epithelial integrity) [13], *IL10* and *STAT3* (cytokine signaling) [14], *NCF4* and *SOD2* (oxidative stress) [15], and *TNFSF15* and *IL2RA* (lymphocyte function) [16]. Here, we discuss the functions of autophagy-related CD susceptibility genes, the specific types of autophagy—xenophagy, ERphagy, and mitophagy—shown to be involved in CD inflammation, and the impact of dysfunctional autophagy on innate immunity in the gut mucosa.

## 2. Innate Immunity in the Gut Mucosa

The gastrointestinal tract is the interface between self and 100 trillion bacteria and luminal antigens. The intestinal epithelium is a single layer of cells tasked with forming a semi-permeable, physical barrier allowing the absorption of nutrients and immune sensing, while blocking harmful pathogens from entry into the underlying lamina propria. The intestinal epithelium is comprised of distinct cell types differentiated from Lgr5^+^ intestinal stem cells (ISCs), including absorptive enterocytes (colonocytes in the colon), and secretory cells, such as goblet and Paneth cells. Goblet cells and Paneth cells play an important role in innate immunity and intestinal homeostasis via their secretory functions. Goblet cells are located in the small intestine and colon and secrete gel-forming mucins that polymerize in the mucosal surface to form the mucus layer that serves as a physical barrier against luminal contents. The proper functioning of goblet cells and their secreted mucins are crucial for an effective mucus barrier and protection against luminal microbiota. Paneth cells are located in the small intestine at the base of the crypts of Lieberkuhn interspersed with Lgr5^+^ ISCs. Paneth cells produce and secrete antimicrobial peptides, such as lysozyme, and defensins that modulate the gut microbiota composition, thereby protecting the gut from harmful bacteria and other microorganisms. Paneth cells also produce other niche factors important for Lgr5^+^ ISC homeostasis [17]. Defective Paneth cells can lead to inflammation in the ileum in mouse models [18,19]. Emerging studies also indicate that defective Paneth cells may lead to an imbalance in the gut microbiota and an increased susceptibility to bacterial invasion and inflammation [19,20]. Moreover, CD risk alleles related to autophagy converge in causing impairment of the secretory functions of goblet and Paneth cells [21,22].

In addition to barrier function, intestinal epithelial cells, along with dendritic cells (DCs) and macrophages, play major roles in the development and maintenance of tolerance toward luminal antigens and commensal microbiota or activation of immune responses. It is well-established that intestinal epithelial cells serve as non-classical antigen presenting cells via major histocompatibility complex class II molecules (MHC II) to elicit microbiota specific immunity [23]. Given their proximity to the intestinal lumen, this antigen presenting ability of epithelial cells serves as an early first-line response to promote homeostasis. Intestinal DCs are antigen presenting cells in the intestinal lamina propria, Peyer’s patches, and draining lymph nodes that continuously surveil luminal antigens via migration into the epithelium [24]. Depending on the antigens encountered, DCs have the ability to promote tolerance through the generation of retinoic acid and FoxP3^+^ T regulatory (Treg) differentiation or to promote the T effector cell function, in particular Th17 cells [25]. Importantly, it has been demonstrated that goblet cells can transport luminal antigens to underlying lamina propria DCs [26]. Residential macrophages of the lamina propria support tolerance to gut microbiota dependent on routine sensing, responsiveness to IL-10, and the presence of TGFβ to induce FoxP3^+^ Tregs [27]. These resident intestinal macrophages remain refractory to inflammatory stimuli and present antigens via MHC II to promote tolerance. Upon breach of the intestinal epithelium, or other inflammatory signaling, an influx of inflammatory macrophages that originate from circulating Ly6+ monocytes ensues. These inflammatory macrophages recognize and eliminate pathogens, such as invading bacteria, that translocate past the epithelium via strong bactericidal activity and clearance by phagocytosis. Intestinal macrophages also produce pro-inflammatory cytokines IL-1, IL-6, and TNF and express costimulatory molecules, such as CD40 [27].

Here, we discuss the importance of autophagy in the functioning of the specific intestinal cells (Paneth cells, goblet cells, DCs, and macrophages) involved in gut innate immunity and their relevance to CD. In addition to innate immunity, the role of autophagy in adaptive immunity is also crucial for intestinal homeostasis and has been recently reviewed [28,29].

## 3. Autophagy

Autophagy is a cellular process that plays a critical role in maintaining cellular homeostasis by degrading and recycling intracellular components, such as damaged organelles and misfolded proteins, and by providing cells with energy during times of nutrient deprivation. The term autophagy comes from the Greek words “auto” meaning self and “phagy” meaning eating, and refers to the process by which cells break down their own components [30]. During autophagy, cells sequester damaged or unwanted cellular components within a membrane-bound structure called an autophagosome. The autophagosome then fuses with a lysosome, a specialized cellular organelle that contains enzymes capable of breaking down and recycling cellular components [30]. Autophagy is also involved in regulating the host response to bacteria and other microorganisms in the gut. Dysregulation of autophagy can lead to impaired bacterial clearance and an increased susceptibility to infections. Dysregulation of autophagy has been implicated in a number of diseases, including cancer, neurodegenerative disorders, infections, autoimmune diseases, metabolic disorders, and CD [30,31].

**Figure 1:**
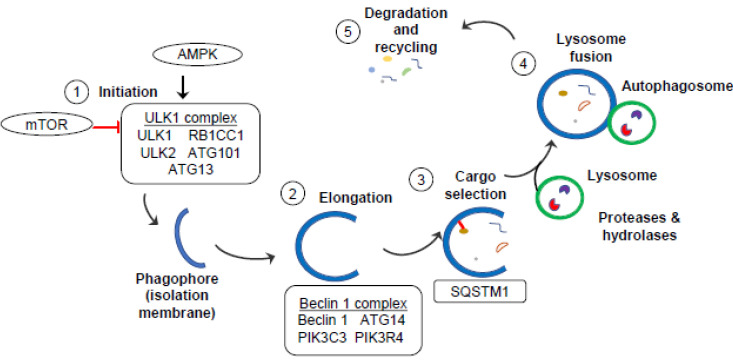
Autophagy pathway. (1) mTOR inhibits the initiation of autophagy, whereas stress (such as bacterial infection or reactive oxygen species) activates AMPK that promotes activation of the ULK1 complex leading to the formation of a phagophore. (2, 3) Formation of the Beclin 1 complex facilitates elongation of the phagophore and cargo engulfment into a fully formed autophagosome. (4, 5) Lysosomes containing digestive enzymes (proteases and hydrolases) fuse with the autophagosome where the cargo is degraded.

Autophagy is a complex process involving several steps: initiation, elongation, cargo selection, and degradation/recycling (Figure 1) [32,33]. 

Initiation: the autophagy pathway is tightly regulated by several signaling pathways, including the mechanistic target of rapamycin (mTOR) pathway, which inhibits autophagy in the presence of nutrients and growth factors, and the AMP-activated protein kinase (AMPK) pathway, which stimulates autophagy in response to energy depletion [34]. The first step of autophagy is the formation of a membrane structure called the isolation membrane or phagophore. This is initiated by the activation of a complex of proteins, called the ULK1 (Unc-51-like kinase 1) complex, composed of ULK1 (also known as autophagy-related 1 (ATG1)), ULK2, ATG13, RB1-inducible coiled coil 1 (RB1CC1), and ATG101. ULK1 regulates the transmembrane protein ATG9, which acts as a phagophore initiator by recruiting lipids from cellular sources, such as the endoplasmic reticulum (ER), mitochondria, and endosomes [35]. 

Elongation: The Beclin 1 complex is a key regulator of phagophore elongation via its regulation of phosphatidylinositol 3-phosphate (PI3-P) de novo lipid production. The Beclin 1 complex is composed of Beclin 1, ATG14, phosphatidylinositol 3-kinase catalytic subunit type 3 (PIK3C3), and phosphatidylinositol 3-kinase regulatory subunit 4 (PIK3R4). Beclin1 and ATG14 are phosphorylated by the ULK1 complex, promoting the formation of PIK3C3 complexes to convert phosphoinositide (PI) to PI3-P lipids. Microtubule-associated light chain 3 (LC3) is processed by ATG4 to produce LC3I. This process involves the synthesis of LC3 as LC3I, which consists of a cytosolic N-terminal domain and a C-terminal glycine residue. LC3I is then activated through the proteolytic cleavage of its C-terminal region by the cysteine protease ATG4. This cleavage exposes the glycine residue that is crucial for the lipidation step. Then, ATG3 and ATG7 conjugate phosphatidylethanolamine (PE) to LC3I, producing the lipidated form, called LC3II, that is bound to the forming autophagosome membrane [36]. Elongation is further directed by ATG12, ATG5, and ATG16L1 until the autophagosome is closed with two distinct inner and outer lipid membrane bilayers. 

Cargo selection: As the phagophore is forming, cytoplasmic material can be indiscriminately engulfed or specific cargo can be selected via adapter molecules. A well-characterized adaptor molecule involved in selecting cargo is p62/Sequestosome 1 (SQSTM1). SQSTM1 promotes degradation of polyubiquitinated cargo, such as protein aggregates or damaged mitochondria, via its dual binding to ubiquitin chains and to LC3II [37]. In this way, SQSTM1 links ubiquitinated proteins to the autophagic machinery for incorporation into the forming vesicle. 

Degradation and recycling: The autophagosome is trafficked along microtubules to a lysosome where small GTPases, such as Rab7, SNAREs, and the endosomal sorting complex required for transport (ESCRT), facilitate the fusion of the outer autophagosome membrane with the lysosome. This is now called an autolysosome. The acidic lysosomal components degrade the cargo and adapter molecules, such as SQSTM1. LC3II can be converted back to LC3I and the degraded contents are recycled back into the cytosol or used to fuel metabolic pathways, thereby promoting cell survival.

## 4. Autophagy-Related CD Susceptibility Genes

Several CD susceptibility genes converge on the autophagy pathway. Dysregulation of autophagy has been implicated in the pathogenesis of CD, and genetic studies have identified *ATG16L1*, *IRGM*, *NOD2*, *LRRK2*, *ULK1*, *ATG4*, and *TCF4* as susceptibility genes for this disease [38] (Table 1). These susceptibility alleles are thought to impair the autophagy process, leading to an accumulation of damaged cellular components, inefficient intracellular bacterial recognition and removal, and increased inflammation in the gut [38]. In addition to their roles in autophagy regulation, these genes have also been shown to play a role in the regulation of the immune response and inflammation [39].

### 4.1. ATG16L1

ATG16L1 (Autophagy-Related 16 Like 1) was the first identified autophagy-related CD susceptibility gene. ATG16L1 functions as a scaffold protein that helps to assemble the autophagy machinery, and is required for the formation of autophagosomes [31]. The *ATG16L1* T300A mutation is associated with an increased risk of developing CD, and several studies have suggested that these mutations may impair autophagy in intestinal epithelial cells and immune cells, contributing to the development of this disease [31]. The T300A variant in ATG16L1 results in aberrant Paneth cell architecture and disordered granularity [20]. Some studies have found that ATG16L1 deficiency leads to impaired autophagy-mediated clearance of intracellular bacteria in intestinal epithelial cells, resulting in increased bacterial load and the activation of inflammatory signaling pathways [31,38]. Further, *ATG16L1* mutations reduce the expression of antimicrobial peptides in intestinal epithelial cells, impairing the ability of these cells to defend against pathogenic bacteria [31].

### 4.2. IRGM

IRGM (Immunity-Related GTPase M) has been shown to interact with components of the autophagy machinery, such as ULK1 and Beclin 1, to promote the formation of autophagosomes in response to infection with intracellular pathogens [38,40,41]. IRGM-deficient mice have impaired clearance of intracellular bacteria in intestinal epithelial cells, leading to increased inflammation and tissue damage [42]. IRGM dampens inflammatory signaling via selective autophagy of the Nucleotide-binding domain, Leucine-rich Repeat-containing Family, and Pyrin Domain Containing 3 (NLRP3) inflammasome, thereby inhibiting its activation and downstream signaling [43]. In addition, IRGM1-deficient mice demonstrate Paneth cell abnormalities and elevated susceptibility to intestinal inflammation [44]. *IRGM* SNPs rs1000113, rs11747270, rs9637876, rs13361189 and rs180802994 are associated with CD [45]. Some studies have reported that *IRGM* SNPs may increase fecal lactoferrin (inflammatory biomarker) from the ileum and TNF in whole blood in humans with CD [40]. Further studies are warranted to explore more functional alterations due to *IRGM* SNPs in CD.

### 4.3. NOD2

*NOD2* (Nucleotide-Binding Oligomerization Domain-Containing Protein 2) encodes a protein involved in the regulation of the immune system and the maintenance of the intestinal barrier function [46,47,48]. NOD2 is an intracellular receptor that recognizes muramyl dipeptide motifs, a component of the bacterial cell wall, and upon activation elicits responses to eradicate invading pathogens including activation of NFκB inflammatory signaling. Recent studies have shown that NOD2 interacts with several proteins involved in autophagy, including ATG16L1 and IRGM, to recruit autophagy machinery to the cellular site of invading bacteria and incorporate bacteria into the forming autophagosome. This has been shown to be important in intestinal epithelial cells at the interface with the gut microbiome [38,49]. Genetic variation in *NOD2* accounts for 20% of CD genetic risk, with three common variants, R702W, G908R, and L1007fs, particularly associated with ileal CD [50]. *NOD2* mutations impair NFκB and autophagic responses to intracellular bacteria, leading to the accumulation of bacteria in intestinal epithelial cells [51]. *NOD2* mutations have also been shown to reduce the expression of ATG16L1 in intestinal epithelial cells as an additional mechanism whereby autophagy-mediated clearance of intracellular bacteria is impaired [52]. Furthermore, NOD2 has been implicated in the Paneth cell-mediated responses against intestinal bacteria-induced inflammation [53].

### 4.4. LRRK2

LRRK2 (Leucine-Rich Repeat Kinase 2) plays a role in several cellular processes, including autophagy, the immune response, and the regulation of mitochondrial function [54,55]. LRRK2 has been demonstrated to regulate the formation of autophagosomes in intestinal epithelial cells and to maintain functional Paneth cells in CD [56]. LRRK2 plays a role in regulating the formation of autophagosomes by interacting with several proteins involved in this process. One such protein is Rab29, which is a small GTPase that regulates the recruitment of the autophagy-related protein ATG9 to the site of autophagosome formation [57]. In addition, LRRK2 has been shown to phosphorylate Rab29, which enhances its ability to recruit ATG9 to the site of autophagosome formation [57], and to phosphorylate ULK1, enhancing its activity and promoting the initiation of autophagy [58].

Mutations in the *LRRK2* gene have been identified as a genetic risk factor for several diseases, including Parkinson’s disease and CD [54,59]. *LRRK2* mutations impair the autophagic response to intracellular bacteria in intestinal epithelial cells, leading to an accumulation of bacteria and the activation of inflammatory signaling pathways [60]. In addition to its role in autophagy regulation, LRRK2 has also been shown to regulate the immune response and the expression of several toll-like receptor (TLR)-mediated inflammatory cytokines and chemokines [61]. *LRRK2* mutation (M2397T polymorphism), associated with CD, alters CD14^+^ monocyte-derived type II interferon (IFN) response [62]. Moreover, different variants of *LRRK2* are linked to CD and Parkinson’s disease. In CD, the exact mechanisms by which LRRK2 variants contribute to CD pathology are not yet fully understood. However, it is speculated that LRRK2 variants, such as *LRRK2* G2019S, may impact immune responses, and intestinal barrier functions, or alter gut microbial composition [63,64,65]. In addition, a mutation in the *LRRK2* N2081D allele results in elevated kinase activity while the *LRRK2* R1398H variant increases its enzymatic activity altering LRRK2 functionality in CD and Parkinson’s disease [66]. In Parkinson’s disease, several pathogenic LRRK2 variants, such as G2019S and R1441C/G, have been linked to an increased risk of developing this disease [64,67]. These variants are thought to lead to increased LRRK2 kinase activity, which can contribute to neuronal dysfunction and degeneration. Dysregulated kinase activity may affect various cellular processes, including mitochondrial function, protein degradation pathways, and synaptic transmission, ultimately leading to the development of Parkinson’s disease symptoms [67,68].

### 4.5. ULK1

ULK1 (Unc-51-Like Autophagy Activating Kinase 1) is a protein kinase that plays a critical role in the initiation of autophagy. ULK1 is part of a protein complex known as the ULK1 complex, which also includes ATG13, FIP200, and ATG101 [69]. This complex is activated in response to cellular stress, such as nutrient deprivation or the accumulation of damaged cellular components [69]. Once activated, the ULK1 complex phosphorylates several downstream targets, including the protein Beclin 1, which triggers the formation of autophagosomes [69]. In addition to its role in autophagy initiation, ULK1 also has several other cellular functions, including the regulation of cell growth and differentiation, metabolism, and the response to oxidative stress [70,71,72]. Dysregulation of ULK1 has been implicated in several diseases, including CD, cancer, neurodegenerative disorders, and metabolic disorders [73]. In regards to CD, studies have shown that ULK1 may play a role in regulating the differentiation and function of immune cells [74]. Dysregulation of ULK1 has also been linked to increased intestinal permeability, which can lead to the infiltration of bacteria and other harmful substances into the intestinal tissue, triggering an inflammatory response [75]. The SNP rs12303764 in *ULK1* is associated with CD [74].

### 4.6. ATG4

ATG4 (Autophagy-related protein 4) is a cysteine protease that plays a critical role in the regulation of autophagy via processing of LC3 [76]. Missense variants in *ATG4C* associated with CD are N75S, R80H, C367Y, K371R, R389X, and a frameshift mutation specifically enriched in Finland (1:62819215:C:CT) [9]. Four of these are truncating variants, suggesting loss-of-function variants in *ATG4C* increase the risk of CD. Several studies have suggested that dysregulation of ATG4-mediated processing of LC3 may contribute to the development and progression of CD. A study reported that mice lacking ATG4B, one of the isoforms of ATG4, had impaired autophagy and were more susceptible to the development of inflammation [77]. It has also been reported that ATG4B is downregulated in the intestinal mucosa of patients with CD [78], suggesting that dysregulation of ATG4-mediated autophagy may contribute to the development of this disease.

### 4.7. TCF4

TCF4 (Transcription Factor 4) is a member of the basic helix--loop--helix (bHLH) family of transcription factors that play an important role in the regulation of gene expression and cell survival [79,80]. As a Wnt signaling pathway transcription factor, TCF4 plays an important role in the health of Paneth cells and production of antimicrobial peptides Defensin-5 and Defensin-6 in a Wnt-pathway-dependent manner [81]. TCF4 deficiency leads to impaired autophagy in intestinal epithelial cells, resulting in the accumulation of intracellular bacteria and the activation of inflammatory signaling pathways [82]. SNP rs3814570 in the *TCF4* promoter region is associated with ileal CD, with the strongest association in patients with stricturing disease [83].

## 5. Types of Autophagy Linked to CD Pathogenesis

Autophagy is grouped into three major categories: microphagy (microautophagy), chaperone-mediated autophagy (CMA), and macrophagy (macroautophagy). These types of autophagy are mechanistically distinct from each other, but the end result of all three is delivery of cargo to the lysosome for degradation.

In microautophagy, the lysosome directly engulfs and degrades cellular components through the invagination of the lysosomal membrane, which surrounds the targeted cellular component and sequesters it inside the lysosome [84]. Microphagy is a mechanism by which peroxisomes, portions of the nucleus (nuclear pores, chromatin, and nucleoli), ribosomes, and glycogen are degraded [84,85,86,87,88,89]. CMA is a selective form of macroautophagy that involves the direct recognition and targeting of specific proteins for degradation by the lysosome [90]. The process of CMA involves a series of steps. First, the proteins marked by the KFERQ motif are recognized by HSC70 in the cytosol. HSC70 then assists in unfolding and translocating the targeted proteins across the lysosomal membrane by interacting with LAMP1. Once inside the lysosome, the proteins are degraded into smaller peptides, which can be further processed for recycling or energy generation [91]. There are several types of CMA: constitutive CMA, induced CMA, and atypical CMA. Constitutive CMA is the basal CMA that occurs in most cells. It targets proteins with the CMA recognition motif, which is a specific amino acid sequence found in certain proteins [92]. Constitutive CMA is important for the turnover of normal cellular proteins and the removal of damaged or misfolded proteins [93]. Induced CMA is a stress-responsive form of CMA that is activated by nutrient deprivation, oxidative stress, or protein misfolding. Induced CMA can target a wider range of proteins than constitutive CMA, including those without the CMA recognition motif. This allows it to selectively remove damaged or misfolded proteins that may accumulate during stress [93]. Atypical CMA is a form of CMA that targets proteins that are not recognized by the typical CMA machinery. Atypical CMA is less well understood than constitutive and induced CMA, but it is thought to play a role in the turnover of specific proteins, such as those involved in synaptic transmission [94]. Dysregulation of microphagy or CMA have been implicated in various diseases, including neurodegenerative disorders and cancer [95,96], but have not yet been implicated in IBD.

**Figure 2:**
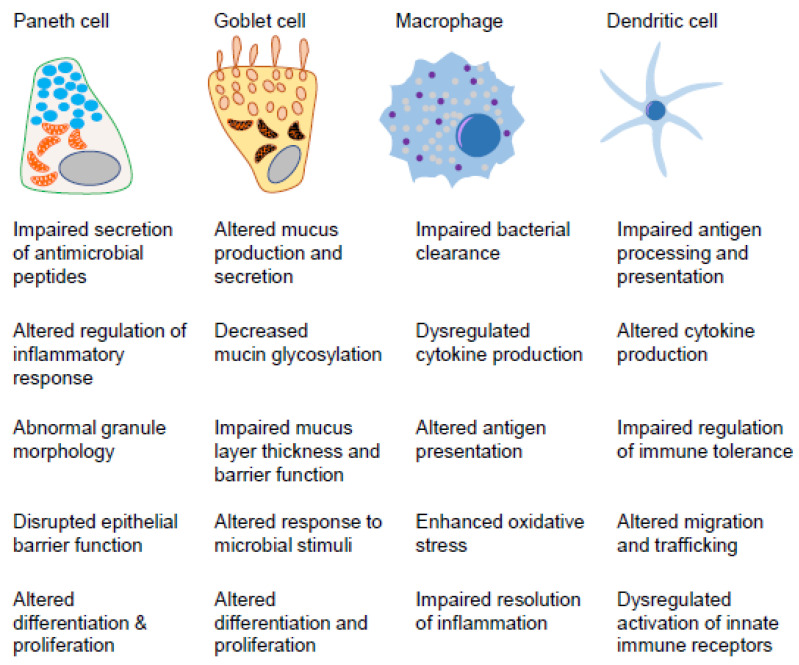
Functional alterations during autophagy impairment in cells mediating mucosal innate immunity. Known functional alterations associated with autophagy dysfunction in intestinal innate immune cells relevant to CD.

Macroautophagy is the most studied type of autophagy that is linked to CD pathogenesis, and is a complex, multi-step process involving numerous proteins (discussed in Section 3). There are several types of macroautophagy that fall within non-selective and selective categories. In non-selective macroautophagy, the autophagosome forms indiscriminately around cytoplasmic components, including proteins, lipids, and organelles. This type of macroautophagy is important for bulk degradation and recycling of cellular components during nutrient deprivation or stress. In contrast, selective macroautophagy involves the specific recognition and degradation of certain cellular components, such as damaged organelles or intracellular pathogens [97]. There are several types of selective macroautophagy, including xenophagy, ER-phagy, mitophagy, ribophagy, and ferritinophagy. Current studies have implicated dysfunctional xenophagy, ER-phagy, and mitophagy in intestinal inflammation. Of note, it has emerged across collective studies that specific intestinal cell types involved in mucosal innate immunity (macrophages, DCs, Paneth cells, and goblet cells) are susceptible to dysfunctional xenophagy, ER-phagy, or mitophagy (Figure 2).

### 5.1. Xenophagy

Xenophagy is a specialized form of autophagy that involves the degradation and elimination of invading microorganisms, such as bacteria, viruses, and parasites [98,99]. During xenophagy, the microorganisms are engulfed by an autophagosome and degraded in the autolysosome. Xenophagy is an important innate immune defense mechanism. It is activated by specific cellular receptors, such as TLRs, NOD-like receptors (NLRs), pyrin domain containing 3 (NLRP3), RIG-I-like (RLRs), and C-type receptors (CLRs), that recognize the presence of microbial components, such as bacterial cell wall components or viral nucleic acids [100,101]. TLRs are expressed on the surface of immune cells and basolaterally on intestinal epithelial cells and recognize specific pathogen-associated molecular patterns (PAMPs) on the surface of pathogens. TLR activation triggers downstream signaling cascades that can activate xenophagy and other immune responses [102]. NLRs, including NOD2, are cytosolic pattern recognition receptors that surveil the intracellular environment and activate inflammatory cascades and inflammasomes in response to infection [103,104]. NLRP3 recognizes various PAMPs and danger-associated molecular patterns (DAMPs), including bacterial cell wall components and damaged or dying host cells, respectively [105]. In addition to NLRP3 and TLRs, other receptors involved in xenophagy include RLRs, which recognize viral RNA, and the cGAS-STING pathway, which senses cytosolic DNA. These receptors activate signaling pathways that converge on the autophagy machinery to promote the clearance of invading pathogens and pathogenic DNA [106]. Dysregulation of xenophagy has been linked to various infectious diseases, including tuberculosis, Salmonella infection, and viral infections such as influenza and HIV [101,107].

Xenophagy has been shown to play a critical role in modulating the function of macrophages and DCs in several ways. Xenophagy regulates the production of proinflammatory cytokines, such as interleukin-1 beta (IL-1β), interleukin-6 (IL-6), and tumor necrosis factor-alpha (TNF-α) in macrophages and DCs [108,109]. Xenophagy can regulate inflammasome activation and subsequent IL-1β production by controlling the degradation of bacterial or viral components and regulating the activation of signaling pathways downstream of cytokine receptors in response to bacterial infection [110]. Xenophagy can dampen the production of type I IFNs in response to viral infection by restricting the replication of viral pathogens in infected cells. Conversely, xenophagy can enhance the activation of interferon-stimulated genes (ISGs), including ISG15, UbE1L, UbcH8, and Herc5 [111,112], important for the innate immune response. Xenophagy can modulate cytokine receptor signaling by regulating the turnover of cytokine receptors. For example, xenophagy can mediate the degradation of the interleukin-1 receptor (IL-1R) and the type I IFN receptor (IFNAR), leading to the downregulation of IL-1β and type I IFN signaling, respectively [113]. Xenophagy also plays a critical role in modulating antigen presentation in macrophages and DCs [114]. Xenophagy can facilitate the processing of antigens from intracellular pathogens for presentation on MHC II molecules, leading to the activation of CD4+ T cells [113]. Xenophagy can also regulate the turnover of MHC II molecules, which can impact the efficiency of antigen presentation [115]. Through these mechanisms, xenophagy plays a critical role in modulating functional responses of macrophages and DCs, impacting the innate immune response to intracellular pathogens.

Multiple studies have suggested that defects in xenophagy may contribute to the development and progression of CD. Studies have shown that ATG16L1-, ATG4-, ULK1-deficient mice exhibit defective xenophagy and are more susceptible to bacterial infections in the gut [116,117,118]. Studies have also shown that *IRGM* expression is reduced in patients with CD [119], and that IRGM-deficient mice exhibit defective xenophagy and are more susceptible to bacterial infections in the gut [44]. In addition, mice lacking IRGM have been shown to exhibit defective Paneth cells accompanied by intestinal injury [120]. Loss of functional mutations in *NOD2*, associated with CD, result in decreased response to invading bacteria and abnormal immune responses driving chronic intestinal inflammation [103,104]. Mutations in *LRRK2* have been associated with an increased risk of developing Parkinson’s disease, and some studies have suggested that LRRK2 may also play a role in regulating xenophagy in the gut [121].

### 5.2. ERphagy

ERphagy is a selective form of autophagy that involves the degradation of endoplasmic reticulum (ER) by autophagy. The ER is a complex network of interconnected tubules and flattened sacs that plays a critical role in protein synthesis, lipid metabolism, and calcium homeostasis. ERphagy is initiated by the recognition and isolation of a specific region of the ER by a protein called FAM134B, which acts as an ER-phagy receptor [122]. FAM134B binds LC3II and promotes the incorporation of the targeted ER region into an autophagosome that is forming.

ER stress occurs when there is an imbalance between protein folding demand and capacity in the ER [123]. ER stress can be induced by a variety of factors, including inflammation, oxidative stress, and genetic mutations. In CD, chronic inflammation in the intestinal mucosa can induce ER stress in various cells, including Paneth cells and goblet cells [124]. In CD, ER stress in Paneth cells has been shown to impair the secretion of antimicrobial peptides, leading to dysbiosis and increased susceptibility to bacterial infections [20,125]. Similarly, ER stress in goblet cells has been shown to impair mucus secretion and increase susceptibility to bacterial adhesion and invasion in CD [125,126]. The unfolded protein response (UPR) is a cellular stress response pathway that is activated in response to ER stress [127]. The UPR is initiated by three transmembrane proteins: PERK, ATF6, and IRE1. In CD, the UPR pathway is activated in Paneth cells and goblet cells, leading to increased expression of genes in humans (HSPA5 encoding BIP/HSP70, *DERL1*, *EDEM*, *ATF6*, *XBP1*, and *IRE1*) that are involved in protein folding, degradation, and secretion [124].

ERphagy plays an important role in dampening ER stress and maintaining ER homeostasis by removing damaged or excess ER. Dysfunction of ERphagy has been implicated in several diseases, including CD, neurodegenerative disorders, and cancer [128,129]. ERphagy is regulated by several key proteins, including X-box binding protein 1 (XBP1) and ATG16L1 [130,131]. XBP1 is a transcription factor that regulates the expression of genes involved in protein folding, secretion, ER stress response, and UPR [132,133]. Dysregulation of the UPR has been implicated in the pathogenesis of CD [134], and studies have suggested that XBP1 plays a role in the regulation of autophagy in response to ER stress [135]. Mice with deletion of Xbp1 in the intestinal epithelium (*Xbp1^ΔIEC^*) developed ER stress, Paneth cell impairment, and mild-to-moderate spontaneous ileitis [18]. Loss of autophagy via crossing to mice with deletion of Atg7 in the intestinal epithelial cells (*Atg7*/*Xbp1^ΔIEC^*), resulted in unresolved ER stress and a worsening of ileitis compared to *Xbp1^ΔIEC^* mice [18]. Later studies demonstrated that prolonged activation of the UPR driving accumulation of IRE1α aggregates contributed to the development of transmural ileitis when not dampened by autophagy [136]. Studies have shown that XBP1 plays a role in the regulation of ERphagy by promoting the expression of FAM134B, the ERphagy receptor, and that ATG16L1 interacts with FAM134B and promotes ERphagy [130,137]. Similar to Paneth cells, impairment of ERphagy in mice by ATG16L1, IRGM1, or NOD2 deficiency results in goblet cell mucus secretion defects and compromised barrier function [44,126,138]. ER stress unresolved by ERphagy may be especially deleterious in intestinal secretory cells, such as Paneth cells or goblet cells, due to their high reliance on ER for their protein secretory functions.

### 5.3. Mitophagy

Mitophagy is a specialized form of autophagy that involves the selective degradation and recycling of damaged or dysfunctional mitochondria, the energy-producing (ATP) organelles within cells. Damaged or dysfunctional mitochondria can produce harmful reactive oxygen species and contribute to cellular dysfunction and disease [139]. During mitophagy, mitochondria are targeted for degradation by specific receptors, such as Nix/Bnip3L or PINK1 recruitment of Parkin, and engulfed by the autophagosome. Dysregulation of mitophagy has been linked to various diseases, including neurodegenerative disorders, IBD, cancer, and metabolic disorders [140,141,142].

Similar to ER stress, studies investigating mitochondrial stress in the intestine have revealed the role of mitochondrial health as being crucial to Paneth cell health and functionality. A recent study induced intestinal epithelial cell-specific mitochondrial dysfunction via deletion of the mitochondrial chaperone protein Prohibitin 1 [19]. The mice developed spontaneous ileitis that was preceded by mitochondrial dysfunction in all epithelial cells, crypt cell death, and defects in Paneth cells. Importantly, IBD patients exhibit intestinal epithelial mitochondrial dysfunction and decreased expression of PHB1 [141]. Paneth cell abnormalities, including loss of antimicrobial peptide expression, and ileitis could be ameliorated in PHB1 deficient mice by the administration of a mitochondrial-targeted antioxidant, Mito-Tempo, suggesting that mitochondrial reactive oxygen species contributed to disease progression in this model of intestinal epithelial cell mitochondrial dysfunction [19]. A specific role of Paneth cell mitochondrial dysfunction in driving ileitis was demonstrated using mice with Paneth cell-specific deletion of PHB1 [19]. Further studies in these mice revealed a loss of Nix/Bnip3L-mediated mitophagy in the intestinal epithelium during PHB1 deficiency [143], suggesting mitochondrial dysfunction coupled with inefficient removal by mitophagy.

Collectively, these results identify Paneth cells as highly susceptible to mitochondrial dysfunction. Deficiency in *Atg16L1*, *Atg7*, *Atg5*, or *Irgm1* in mice causes Paneth cell defects and CD autophagy-related susceptibility genes, or their disease-linked variants (*ATG16L1* T300A, *LRRK2*, *IRGM*), are associated with Paneth cell abnormalities in the ileum [144,145,146]. Recent studies demonstrated mitochondrial impairment in abnormal CD Paneth cells that correlated with *ATG16L1* T300A mutation [147]. Furthermore, *LRRK2* serves as a significant risk factor for both Parkinson’s and Crohn’s disease, conditions associated with impairments in mitophagy and/or mitochondrial health [64]. Mitochondrial health may be especially important in Paneth cells due to their long-lived nature (30–60 days), compared to other intestinal epithelial cells [17], and their extensive ER for secretory functions coupled with the known communication between mitochondria and ER [148]. Future studies are needed to fully understand the complex interplay between mitochondria and ER in the intestine during health and disease and how it impacts specific epithelial and immune cell types.

## 6. Targeting Autophagy as Therapy

Targeting autophagy as therapy is an area of active research in various fields of medicine, including CD, cancer, neurodegenerative disorders, and infectious diseases. Pre-clinical studies have shown that many current CD therapies include mechanisms that involve modulation of autophagy. For example, anti-TNFα drugs, such as infliximab and adalimumab, are used to suppress inflammation in CD patients but have also been shown to induce autophagy through activation of M2-type macrophages in concert with degradation of NFκB [34,149]. Another common IBD therapeutic, thiopurines, are immunosuppressive drugs that also reverse dampened intestinal epithelial cell wound healing during autophagy impairment [150]. Additionally, several drugs, such as rapamycin, torin1, and metformin, that target autophagy are currently being evaluated as potential treatments for CD [151,152]. Although these drugs have shown potential activities as autophagy-inducers in CD patients, their exact mechanisms in altering autophagy are not fully elucidated [152]. Given the heterogenous nature of CD, it is likely that targeting autophagy is most effective in certain patient populations, such as those carrying mutations in autophagy-related genes or those with Paneth or goblet cell defects. As the field moves forward with more personalized approaches using patient-specific genetics and disease phenotypes, targeting autophagy holds promise in treating CD subtypes with autophagy alterations [152].

## 7. Conclusions

Innate immunity in CD is associated with environmental factors, gut microbiota, and genetic susceptibility, including gene mutations converging on autophagy. Autophagy impairment in CD drives the loss of innate immune responses against exogenous stimuli, such as bacteria, and loss of protective pathways contributing to intestinal homeostasis. Current studies have provided important insights into the role of autophagy in DCs, macrophages, Paneth cells, and goblet cells. Future studies will provide a more complete picture as the role of autophagy emerges in other intestinal cell types, such as enteroendocrine cells, tuft cells, intraepithelial lymphocytes, and innate lymphoid cells. These studies will be valuable as autophagy-targeting therapies evolve in the treatment of CD.

## Figures and Tables

**Table 1:** Summary of autophagy-related CD susceptibility genes.

Gene	Functional Relation to Autophagy
*ATG16L1*	Assembly of autophagy machinery, autophagosome formation, and bacterial defense by Paneth cells
*IRGM*	Promotion of autophagosome formation, dampening inflammation via NLRP3, and maintenance of Paneth cell health
*NOD2*	Regulation of immune system, maintenance of intestinal barrier integrity, elimination of invading bacteria via activation of NFκB, and interaction with ATG16L1 and IRGM to form autophagosomes
*LRRK2*	Regulation of autophagosome formation via Rab29, ATG9, and ULK1 interactions, maintenance of functional Paneth cells, and mediation of the immune response through TLRs
*ULK1*	Initiation of autophagy by forming ULK1 complex, regulation of immune cell growth and differentiation, and maintenance of intestinal barrier function
*ATG4*	Regulation of autophagy via processing of LC3 and reduction of inflammation
*TCF4*	Contribution to functional autophagy, regulation of gene expression and survival, and ensuring Paneth cell health

## Data Availability

Not applicable.
